# Teachers’ adoption of an open and interactive e-book for teaching K-12 students Artificial Intelligence: a mixed methods inquiry

**DOI:** 10.1186/s40561-021-00176-5

**Published:** 2021-12-11

**Authors:** Xiangling Zhang, Ahmed Tlili, Keith Shubeck, Xiangen Hu, Ronghuai Huang, Lixin Zhu

**Affiliations:** 1 Beijing Institute of Education, Beijing, China; 2 Smart Learning Institute of Beijing Normal University, Beijing, China; 3 Department of Psychology, The University of Memphis, Memphis, USA

**Keywords:** Interactive, e-books, Artificial intelligence (AI), Technology acceptance model (TAM), Open educational resources (OER), Open educational practices (OEP)

## Abstract

With the rapid development of information technology, e-books have become convenient for students to improve their learning performance, especially when learning complicated concepts. However, research showed that acceptance of e-books by teachers is fragmented, due to several factors including the e-book design. Therefore, this study combined the potential positive impacts of openness and interaction on learning to design an open and interactive e-book for teaching K-12 students AI. It then applied a mixed method to investigate the factors that affect teachers’ acceptance of this open and interactive e-book based on the technology acceptance model (TAM) and interviews. The obtained results showed that teachers’ intention to continue using this e-book is significantly influenced by their perceived usefulness and attitude towards this e-book. Additionally, both the interactive and openness features were very helpful for teachers in using this e-book in their teaching plans. However, some of them raised several concerns like the interactive coding platform should be personalized based on students’ age. The findings of this study could help different stakeholders (e.g., instructional designers, teachers, policymakers) in facilitating the design and adoption of open and interactive e-books.

## Introduction

Technology has been regarded as one of the most essential elements in the advancement of educational systems (Al-Qatawneh et al., 2019), including Electronic Books (e-books), which are a technology-enabled publication form that provides easier and higher quality access to knowledge compared with traditional media, such as printed books (Chen et al., 2013; Lin et al., 2015; Tang, 2021). E-books can be used as learning materials for in-class lectures by navigating and interacting with the provided digital learning content (Hafed et al., 2020; Liu et al., 2020; Turel & Sanal, 2018). They can also be used by students in their out-of-school time in a flipped learning context (Hwang & Lai, 2017). The debate about the effectiveness of e-books is large. For instance, several studies pointed out that e-books reduce cognitive outcomes especially higher order thinking (Sung et al., 2019) and can easily distract students (Kelley & Warburton, 2011). Other studies, on the other hand, pointed out that e-books can enhance students’ access to information, and also help revolutionize the processes of reading, analyzing, and researching (Blummer & Kenton, 2020; Rothman, 2006). Therefore, they have received overwhelming acceptance, especially in higher education (Biranvand & Khasseh, 2014; Khalid et al., 2017), as they not only make great economic sense for consumers (e.g., students and learning organizations), but they also make pedagogical sense for learning in general (Khalid et al., 2017).

While several studies investigated the variables that impact users’ intention usage of e-books from the perspective of students (Beimers, 2014; Biranvand & Khasseh, 2014; Ebied & Rahman, 2015; Khalid et al., 2017; Lin et al., 2019; Liu et al., 2020), limited studies have examined the factors that impact the intention of using e-books from the perspective of teachers, as they also can incorporate them in their teaching strategies and materials. Thus, this study examines the adoption level of e-books within teachers based on the Technology Acceptance Model (TAM; Davis, 1989). TAM is one of the most widely used models to identify to what extent several factors influence the continuance intention of using e-books (Jin, 2014; Ngafeeson & Sun, 2015; Shin et al., 2018), among other technologies.

E-books have covered many disciplines, including languages, math, science, and music, but less focus has been paid to develop e-books for Artificial intelligence (AI) as a subject (Baek & Monaghan, 2013). Additionally, despite the numerous advantages of e-books, only a few are developed for primary and middle school students. Therefore, this study specifically investigates K-12 teachers’ acceptance of an e-book that is designed for teaching AI. TAM provides a framework for explaining how to reinforce teachers’ continuance intention to use a given technology in K-12 settings (Granić & Marangunić, 2019). Particularly, this study investigates how interactivity and openness within e-books might influence teachers’ acceptance of e-books. To the best of our knowledge, no study harnessed the power of interactivity and openness to enhance the design and acceptance of e-books.

## Literature review

### E-books in education

Books play an important role in the teaching/learning process (West et al., 2010) and have been the catalyst for knowledge transmission. The rapid development of information technology has shifted the design of books from printed to electronic. Consequently, multiple types of e-book appeared which promoted the evolution and acceptance of e-books (Bozkurt & Bozkaya, 2015). E-books are a digital version of printed books (Rao, 2003), and interactive e-books are regarded as an advanced version and enhanced extension of digital books (Bozkurt & Bozkaya, 2015).

Despite that e-books have been frequently developed for different subjects, such as mathematics (Malathi & Rohani, 2010; Turel & Sanal, 2018), English (Carol et al., 2019; Jongyun et al., 2021), and nursing (Liu et al., 2020), very limited interactive e-books have been developed for teaching AI (Baek & Monaghan, 2013). Although AI is frequently used and mentioned in the media, there is still a significant lack of understanding it (West & Allen, 2018). As children gain exposure to and increase their understanding of AI technologies, their reasoning about these technologies becomes more thoughtful and nuanced (Druga et al., 2017). However, educators lack knowledge on how to teach teenagers AI (Ting-Chia et al., 2021), and there is a tremendous need for this to happen quickly, especially during the COVID-19 pandemic (Yao et al., 2021). In this context, several initiatives worldwide have been launched to help students learn AI, at an early age (Ali et al., 2019; Williams et al., 2019, 2021). For instance, to prepare both students and teachers for the AI era, Williams et al. (2021) developed an AI curriculum for primary and secondary schools and trained teachers before they conduct an AI course. The results showed that the teachers felt prepared after the training and their students were engaged in the course.

In line with this, this study focuses on developing an e-book for teaching K-12 Chinese students AI. The interactive e-book is structured according to the “Five Big Ideas in AI” developed by the Association for the Advancement of Artificial Intelligence (AAAI) and Computer Science Teachers Association (CSTA) for K-12 students, namely: Perception, Representation & Reasoning, Learning, Natural Interaction and Societal Impact (Touretzky, 2021). Each big idea includes a set of concepts and skills, as well as different learning objectives.

### Design and interaction within e-books

There are four major interaction levels within interactive e-books which depend on the degree of interactivity of users’ involvement in an instructional activity (Bozkurt & Bozkaya, 2015). The description of the four levels is in the following (Bozkurt & Bozkaya, 2015): Level-1 implies that users are passive, where they act solely as information receivers. Level-2 implies that users have limited participation, such as making simple responses to instructional cues. Level-3 implies that users have complex participation and make a variety of responses to instructional cues. Level-4 implies that users can experience real-time participation, including being involved in a life-like set of complex cues. Interactive e-books provide different benefits for students, such as being easily customized according to their reading style or also annotation support, which means that students can edit, add notes or highlight specific text without harming the original work (Bozkurt & Bozkaya, 2015). Bozkurt and Bozkaya (2015) emphasized that interactive e-books provide not only a reading experience but an “e-reading experience”, which includes cognitive, sensorial, and physical interactions. Worm (2013) found that for simple concepts, learning performance stays consistent between e-books and printed books. However, for complicated concepts, interactive e-books can help students learn more efficiently than other forms of books, and improve their performance (Hsiao et al., 2016; Liu et al., 2020; Tsai, 2015). For instance, Liu et al. (2020) showed that students who used the interactive electrocardiogram (EKG)-focused e-book were more motivated to learn complicated EKG concepts and had better learning achievement compared to their peers who used printed books. Despite that interactivity is an essential element for successful learning (McIsaac & Gunawardena, 1996) and plays an essential role that fulfills many critical functions in the educational process (Anderson, 2003), inappropriate interaction design of e-books can have a negative impact on students’ learning experience, especially for young children (Christ et al., 2019).

Therefore, this study designs different interactive e-book functionalities for teaching AI to Chinese K-12 students based on the above four interaction levels highlighted by Bozkurt and Bozkaya (2015). This interactive e-book also supports interaction among users in virtual environments. As practicing  coding is essential for learning AI especially for deeply understanding of various models and complex concepts, this interactive e-book embeds an interactive coding platform so that students can practice their programming skills. To the best of our knowledge, no study has developed a coding platform in their (interactive) e-books which can make the learning experience more immersive.

Furthermore, while open textbooks have started to be adopted worldwide (Hilton, 2016), no study to the best of our knowledge has designed open and interactive e-books to enhance the learning experience. In this context, Wiley and Iiii (2018) mentioned that open educational resources could enable innovative Open Educational Practices (OEP), such as the co-creation and use of teaching materials, where students are more active. This could help to make the learning experience more engaging for students. Therefore, this study designs an open and interactive e-book that engages students in the 5R open activities, namely retain, revise, remix, reuse and redistribute (Wiley & Iiii, 2018).

### Adoption of e-books within teachers

Several studies reported that the adoption of e-books among teachers and students depends on several factors, including the design or functionalities of e-books. For example, Shin et al. (2018) revealed that students’ willingness and computer self-efficacy are positively related to their perceived usefulness and ease of use towards the performance assessment system for e-book production. Ngafeeson and Sun (2015) also explored the effects of technology and system exposure on students’ acceptance of e-books. They found that students’ exposure to a new technology positively affects the acceptance of e-books. Specifically, most of the studies focused on investigating the adoption of e-books among students (Beimers, 2014; Biranvand & Khasseh, 2014; Ebied & Rahman, 2015; Hafed et al., 2020; Jin, 2014; Kennedy & Chiasson, 2016; Khalid et al., 2017), without paying attention to teachers who could also use e-books in their teaching plans and materials. Weng et al. (2018) stated that selecting the appropriate e-book for teachers is a challenge. This could be because teachers rarely adopt technologies due to a lack of technology-related motivation (Iris et al., 2021). In this context, Gökhan et al. (2016) developed a scale for measuring teachers' perception towards Information and Communication Technologies (ICTs). Therefore, it is essential to analyze the factors that influence the continuance intention usage of e-textbooks. Additionally, to the best of our knowledge, no prior studies have investigated teachers’ perceptions towards open e-books; however, several studies investigated teachers’ perceptions towards Open Education Resources (OER), where findings were fragmented (Forgette, 2020; Ganapathy et al., 2015; Ozdemir & Bonk, 2017; Pascual et al., 2018). For instance, Pascual et al. (2018) highlighted the effectiveness of Natural Language Processing Technologies (NLPTs), as Open Educational Resources, in promoting personalized learning language. Tavakoli et al. (2020), on the other hand, pointed out that finding high-quality and relevant OER is time-consuming and not easy for users.

Finally, while several studies investigated the adoption and usage of e-books, most of them focused on higher education (Al-Qatawneh et al., 2019; Biranvand & Khasseh, 2014; Ebied & Rahman, 2015; Hilton, 2016; Jin, 2014; Khalid et al., 2017), without paying too much attention to K-12 education. Therefore, this study investigates the acceptance of a newly developed open and interactive AI e-book within K-12 teachers.

## Research hypotheses

The following hypotheses were investigated to investigate the relationship between the four constructs of TAM within the designed open and interactive e-book, namely: perceived usefulness (PU), perceived ease of use (PEOU), attitude (Att), and continuance intentions (Int).

### Perceived ease of use

Davis and his colleagues defined perceived ease of use as “the degree to which a person believes using a particular system or technology would be free of effort” (Davis, 1989; Davis et al., 1989). In the context of interactive e-books, the perceived ease of use can be defined as the extent to which a user believes that using the designed e-book will not take much effort. Previous studies have shown that perceived ease of use has a positive influence on users’ attitudes and perceived usefulness (Hong et al., 2009). Similarly, perceived ease of use affects the intention to accept an e-book directly or indirectly through perceived usefulness or attitude. Thus, the research hypotheses are proposed in the following:

#### **H1**

Perceived ease of use affects positively the teachers’ perceived usefulness of the open and interactive e-book.

#### **H2**

Perceived ease of use affects positively the teachers’ attitudes towards using the open and interactive e-book.

### Perceived usefulness

Perceived usefulness is defined as the degree to which a person believes that using a particular system or technology would enhance his/her job performance. This means that the user’s subjective assessment of whether using a particular system could improve their performance (Davis et al., 1989). The perceived usefulness of e-books can be described as the extent to which a user thinks it can enhance a teacher’s performance and advance students’ knowledge. Research has revealed that perceived usefulness has an influence on attitude and continued usage intention (Lee et al., 2013). In the literature, it was reported that continuance intention is significantly influenced by perceived usefulness and attitude (Jin, 2014). Thus, we propose the following hypotheses:

#### **H3**

Perceived usefulness affects positively teachers’ attitudes towards using the open and interactive e-book.

#### **H4**

Perceived usefulness affects positively teachers’ intention to continue using the open and interactive e-book.

### Attitude

Attitude is defined as the preference of a user when he/she uses a particular system or technology. It reflects a user’s perception of a positive or negative feeling related to a particular system (Davis, 1989; Davis et al., 1989). The relationship between attitude and continuance intention highlighted in the TAM reflects that attitude is a tendency to evaluate behavior (Wu & Chen, 2017). Previous research has revealed that attitude is the most powerful predictor of the intention to use a given technology (Teo & Zhou, 2014). Jin (2014) reported that attitude towards e-book usage has a significant influence on continuance intention usage of e-books. Thus, we propose the following research hypothesis:

#### **H5**

Attitude affects positively the teachers’ continuance intention of using the open and interactive e-book.

### Continuance intention

**Fig. 1:**
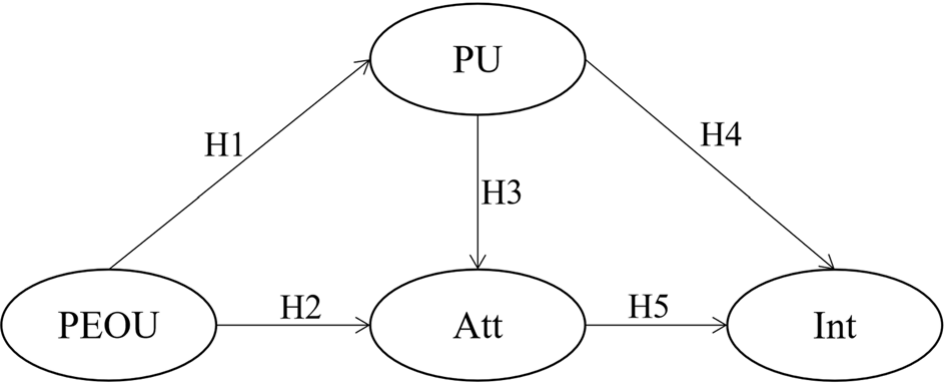
Proposed model for the acceptance of the open and interactive e-book

Intention to use is defined as the cognitive and psychological state of a person’s mind to use a particular system or technology (Davis, 1989; Davis et al., 1989). Jin (2014) found that the continuance intention to use e-books is significantly influenced by users’ satisfaction with e-books. Other studies also revealed that only attitude and perceived usefulness have a significant positive impact on continuance intention (Arteaga & Duarte, 2010; Wu & Chen, 2017). In this study, we investigated the teachers’ intention to use the designed open and interactive e-book. Figure 1 summarizes the hypotheses of this study.

## Study context: open and interactive e-book

### Curriculum outline and design

The learning objective of this open and interactive AI e-book is to introduce AI to Chinese K-12 students, between the age of 9 and 15, in an easy and fun way. The e-book contains six chapters. The first chapter is an introductory chapter to motivate K-12 students about learning AI. The following five chapters focused on teaching AI in line with "Five Big Ideas in AI," namely: Perception, Representation & Reasoning, Learning, Natural Interaction, and Societal Impact (Touretzky, 2021). Each big idea contains concepts and skills. For example, Perception includes several concepts like sensing and processing. Different grades have different learning goals for each concept. For instance, grade-2 students should be able to identify human senses and sensory organs. However, grade 3–5 students should be able to compare human and animal perception.

Williams and Nguyen (2018) stated that school-age children respond better to lessons that involve real-life examples. Therefore, to better explain AI for students, the open and interactive e-book used simple examples from real life that students are familiar with. For instance, to explain the “face recognition” concept (from the perception chapter) to students, the e-book mentioned that:

Recognizing faces is one of the first developed human instincts. Small babies need to recognize the faces of their parents from other faces so they can connect to them. In this context, babies start staring and seeing their parents’ faces (for instance, during breastfeeding) until they memorize them. They can then start seeing the difference between their parents’ faces and unfamiliar faces. Similarly, computers mimic human behavior and intelligence and, instead of using human eyes, they use sensors (cameras) to identify faces.

To increase the motivation of students, the open and interactive e-book further aimed to highlight the relevance of learning AI and how it could be important in real-life (Keller, 1979, 1987, 2000). In this context, for each explained concept, the book gives further stories about how AI is used to combat the COVID-19 pandemic, the most important and discussed topic currently. For instance, the e-book tells the story of using face recognition to detect people that did not respect, for instance, quarantine rules. The motivation behind using the storytelling format is because stories have the potential of engaging students in lessons better than the plain regurgitation of scientific facts (Mohd, 2008).

### Interactivity feature

**Fig. 2:**
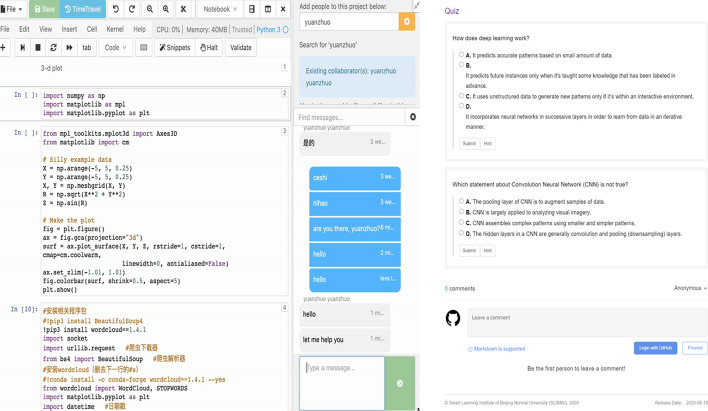
Two learning examples of the interactive e-book

The designed e-book also followed the four-interaction levels (see Sect. 2.2). Particularly, the e-book provides readable content for K-12 students which is Level-1. Additionally, the e-book supports different functionalities like browsing, navigating, annotating, and dragging pages which are Level-2 interactivity. These functionalities aim to give students simple ways of facilitating the learning process. For instance, a student can easily make annotations on specific paragraphs that he/she might be interested in, so he/she can re-read them later on. Level-3 implies that users have complex participation and make a variety of responses to instructional cues. In this study, the interactive e-book provides quizzes for students and gives them hints or feedback in response to their answers (see Fig. 2b). Level-4 implies that students can experience real-time participation, including being involved in a life-like set of complex cues. The highest level of interaction in the developed e-book is between students and the coding platform when they practice coding. To be specific, an online coding platform namely Jupyter (http://jupyter.org) was embedded in the AI interactive e-book. Jupyter is an open-source web-based interactive development environment that can support a wide range of workflows in data science, scientific computing, and machine learning (Terlych & Rodriges, 2020). In this way, students get a hands-on learning experience where they can work in real-time on their programming codes and execute them to see the output, as well as have the possibility to chat with their peers through the chat box (see Fig. 2a).

### Openness feature

The open and interactive e-book was also designed as an Open Education Resource (OER) that allows students to engage in the 5R activities, namely: retain, revise, remix, reuse and redistribute under an open license. For instance, the students can reuse their peers’ programming code to test it out or also to modify it and create other versions out of it. They can also remix their peers’ programming code and redistribute it on other platforms. The students can also reuse the interactive e-book resources, including annotations, quizzes, or figures, to create their own learning materials.

The open and interactive e-book is deployed online (http://actionbook.bnu.edu.cn/ai_handbook/). To validate it, both qualitative and quantitative approaches were used. Specifically, the qualitative approach used focus group discussion (Stewart & Shamdasani, 1998), where several meetings from June 2020 to September 2020 were organized with ten international experts to discuss the appropriateness of the content. Coh-Metrix was also used to further assess the readability of this interactive e-book (Graesser et al., 2004). It is a computational tool that produces indices of the linguistic and discourse representations of a text (McNamara et al., 2014). The produced output values can be used in many different ways to investigate the difficulty, readability and coherence of the mental representation of the interactive book texts. Based on the obtained matrix values, some paragraphs were further revised so they can be easily understood by the students. It should be noted that the validation process of the open and interactive e-book is beyond the scope of this paper.

## Method

Since this experiment was during the COVID-19 pandemic where it was difficult to connect with several teachers in schools, a pilot study was conducted with a limited sample size to have preliminary findings of the developed open and interactive e-book. Two-phase explanatory sequential mixed methods (Tang, 2021) including quantitative method and qualitative method were adopted. The purpose of the quantitative phase is to find out the relationship among variables in TAM to reveal teachers’ continuance intention to use the open and interactive e-book. In this phase, participants completed an instrument after using the e-book. The qualitative phase aimed to seek relevant explanations in participants’ interview data to comprehensively explain and understand quantitative findings.

### Participants

Sixty-five K-12 and college Chinese teachers were recruited through the professional network of the authors, and voluntarily participated in this experiment. These teachers are teaching AI or also other courses that rely on AI, including robotics.

Despite that the open and interactive e-book in this study is mainly for K-12 students, higher education teachers were also invited to further share their long experiences and collect their professional inputs about the open and interactive e-book, hence further contributing to this innovative topic (i.e., open and interactive e-books for teaching K-12 students AI). As shown in Table 1, 76% of the teachers were K-12 teachers, while 24% were college teachers. Additionally, it is seen that 67% of the teachers are teaching Information technology, including algorithms, while 21% and 12% of the teachers are teaching AI and robotics, respectively.

**Table 1:** Demographic information of the participants

Item	Type	Frequency (n = 65)	Percentage
Gender	Female	41	63
	Male	24	37
School level	Primary School (6–12 years old)	26	40
	Middle School (12–15 years old)	18	28
	High School (15–18 years old)	12	18
	College (above 18 years old)	9	14
Teaching Course	Information Technology	41	63
	Robot	9	14
	AI	15	23

### Procedures

As for the quantitative phase, data collection was the first step. A questionnaire about teachers’ perception toward the developed open and interactive e-book was sent to the 65 teachers after using it with their students. Among them, eight teachers gave incomplete answers, hence they were excluded. As a result, the answers from 57 teachers were collected and analyzed in this study.

In order to further understand the effectiveness of variables for teachers' continuance intention to use the open and interactive e-book, interviews were also conducted with ten teachers, who voluntarily agreed to take this interview, to collect their perception about the e-book, especially about the interactivity and openness features. Each interviewee was interviewed for thirty minutes on average. Interviews were audio-recorded and transcribed verbatim. Data analysis was also conducted to ensure the validity and reliability of the findings.

### Instruments

A five-point Likert scale questionnaire ranging from “strongly disagree” to “strongly agree” was adapted and used to investigate the teachers’ adoption level of the newly designed open and interactive e-book (Bhattacherjee, 2000; Venkatesh & Davis, 1996, 2000), based on the four constructs of TAM, namely perceived usefulness (PU), perceived ease of use (PEOU), attitude (ATT) and continuance intention (INT). For example, “I find the AI interactive e-book useful to use with my students” was one of the statements related to the perceived usefulness. Also, “I believe that the digital version of this book is simple to use” was one of the statements related to the ease of use. Two domain experts were invited to validate the questions to ensure their appropriateness for the participants. The construct validity and fitness of the proposed model were examined by evaluating reliability, convergent validity, and discriminant validity.

Semi-structured interviews (Corbin & Strauss, 2008) were conducted to extensively investigate teachers’ continuance intention usage of the open and interactive e-book. A semi-structured interview guide is a list of questions or topics were prepared in advance to be discussed by the interviewer and interviewees. The core question and many associated questions are included in the interview guide. For example, “Were the interactivity functionalities of the e-book helpful in teaching AI? And how so?”; “Was the openness feature of the e-book relevant in your teaching experience?” “What are the challenges you encountered using this e-book in your classes?”.

### Data analysis

Statistical analyses were conducted using R, version 3.63. The six-value summaries (see Table 2) are used to display the data, namely minimum, maximum, median, and the first and third quartiles, as well as the distance between them.

**Table 2:** Description of the six-value summary

Value	Description
Minimum	The lowest data point excluding any outliers
Maximum	The largest data point excluding any outliers
Median	The middle value of the dataset
First quartile (Q1)	The median of the lower half of the dataset
Third quartile (Q3)	The median of the upper half of the dataset
IQR	The distance between the Q3 and Q1

The five hypotheses (see Sect. 2.3) were tested jointly using structural equation modeling (SEM) implemented via partial least squares (PLS) approach which is adequate to analyze the questionnaire data with a small sample size (Chin & Newsted, 1999; Ringle et al., 2012; Wang et al., 2017). Both the structural model (i.e., assessment of the relations between constructs) and a measurement model (i.e., assessment of the reliability and validity of constructs) can be analyzed using these techniques. The test of the structural model includes the R^2^ values, which refers to the extent of variance explained by the independent variables, and path coefficients. This indicates the relationships’ strength between the dependent and independent variables.

A hybrid process of deductive and inductive content analysis was conducted to code the transcripts. First, the researchers individually read the interview data to become familiar with the data. Second, paired researches aggregate relevant codes and recode them into themes.

### Evaluation of reliability and convergent validity

Reliability testing was conducted using Cronbach’s alpha. A construct is considered reliable if it is greater than 0.70 (Hair et al., 1998). Factor loading for each item was also tested, which should be greater than 0.50. Convergent validity was also assessed (Wu & Chen, 2017) based on the following two criteria: (1) Composite Reliabilities (CR), where each construct should be greater than 0.70; and (2) the Average Variance Extracted (AVE) where each construct should be greater than 0.50 (Fornell & Larcker 1981). The AVE, CR, and Cronbach’s alpha of all constructs, as well as the factor loading for each item, are shown in Table 3. All values exceeded the recommended threshold values which indicate that all items were reliable.

**Table 3:** The AVE, CR, and Cronbach’s alpha of all constructs

Construct	AVE	C.R	Cronbach’s α	Factor Loading
*Perceived usefulness*	0.858	0.960	0.959	
PU_1				0.62
PU_2				0.66
PU_3				0.81
PU_4				0.60
*Perceived ease to use*	0.916	0.956	0.956	
PEOU_1				0.55
PEOU_2				0.61
PEOU_3				0.68
PEOU_4				0.59
*Attitude*	0.700	0.823	0.823	
Att_1				0.73
Att_2				0.67
Att_3				0.71
Att_4				0.74
*Intention*	0.865	0.928	0.925	
Int_1				0.53
Int_2				0.63
Int_3				0.74
Int_4				0.62

The reliability and validity of the questionnaire were also tested through confirmatory factor analysis. The indices include Chi-square/degree of freedom (χ2/df), adjusted goodness-of-fit index (AGFI), goodness-of-fit index (GFI), normed fit index (NFI), comparative fit index (CFI) and Tucker Lewis Index (TLI), and root mean square error (RMSE). The model was considered acceptable on condition that χ2/df was less than 3, the AGFI was larger than 0.80, GFI, NFI, CFI, and TLI were all larger than 0.90, and RMSE was lower than 0.10.

Table 4 presents the result of each index, as well as the recommended values. The testing results of all the above values satisfied the criteria of χ2/df (79.569/29 = 2.74), AGFI (0.808), GFI (0.906), NFI (0.908), CFI (0.939), TLI (0.905), and RMSE (0.08). Hence, all indices met the recommended levels, which indicates that the model builds a good fit for the data.

**Table 4:** Goodness-of-fit indicators of the model

Model t indices	Results value	Recommend value
Chi-square/degree of freedom	2.74	≤ 3
Adjusted goodness-of-fit index (AGFI)	0.808	≥ 0.8
Goodness-of-fit index (GFI)	0.906	≥ 0.9
Normed fit index (NFI)	0.908	≥ 0.9
Comparative fit index (CFI)	0.939	≥ 0.9
Root mean square error (RMSE)	0.08	≤ 0.1
Tucker Lewis Index (TLI)	0.905	≥ 0.9

As for the guarantee of the validity and reliability of the interview data, two methods were adopted. First, constant comparison (Patton, 2002) was used to induce themes to guarantee findings were grounded based on the data itself. Second, the member check (Merriam, 1998) method was adopted by sending findings to five participants to validate the results.

## Results

The descriptive statistics of the four constructs (PU, PEOU, ATT, and INT) are shown in Table 5, which can be analyzed in several ways. First, the median and mean of all the four constructs are almost the same and near 5 (far from 1), which indicates that the teachers had higher scores in the four constructs, namely perceived usefulness, perceived ease of use, attitude and continuance intention to use this open and interactive e-book. Second, the data of perceived ease of use is more dispersed than the other three constructs since the IQR is larger. The reason might be because teachers’ standards for perceived ease-of-use vary widely, which means that some teachers did not find the e-book as easy as other teachers.

**Table 5:** The descriptive statistic of the four constructs

	Min	Q1	Median	Mean	Q3	Max	IQR(Q3–Q1)
Perceived usefulness	1	3.5	4	3.908	4.5	5	1.0
Perceived ease to use	1	3.5	4	3.886	5	5	1.5
Attitude	1	3	4	3.763	4	5	1.0
Intention	1	3.5	4	3.904	4.5	5	1.0

The four constructs do not have collinearity is a prerequisite before conducting SEM. In this study, the correlation matrix between the four constructs was analyzed, as shown in Table 6. It was observed that their correlation values were not high, and were less than 0.8, the reference value usually denotes high collinearity (Padilla-Meléndez et al., 2013). Therefore, it can be assumed that the data was satisfied with the prerequisite.

**Table 6:** Correlation matrix

	PU	PEOU	Int	Att
PU	1.00			
PEOU	.41**	1.00		
Int	.30**	.42**	1.00	
Att	.52**	.66**	.52**	1.00

^**^ All correlations were significant at 0.01 (bilateral)

The R^2^ and path coefficients jointly represent how well the data support the hypotheses, as shown in Table 7.

**Table 7:** Effects of the variables on the acceptance of the interactive e-book

	PU	Att	Int
PU		0.343*(H4)	0.381*(H1)
PEOU	0.835*(H5)	0.579*(H3)	
Att			0.536*(H2)
R^2^	77.4%	83%	81.3%

^*^Significant at *p* < 0.01

It is found that attitude was significantly determined by perceived usefulness and perceived ease of use which resulted in an R^2^ of 0.83. This means that the above mentioned two variables (perceived usefulness and perceived ease of use) explained 83% of the attitude. Likewise, the dependent variable continuance intention is significantly determined by attitude and perceived usefulness with an R^2^ of 0.813. In other words, the combined effects of attitude and perceived usefulness explained 81.3% of the variance in continuance intention. Additionally, perceived usefulness was significantly determined by perceived ease of use which explained 77.4% of the variance. In this context, when teachers were asked about what made them satisfied with the open and interactive e-book, one of them mentioned that the interactivity feature brought by the coding platform within the e-book was very helpful to provide a hands-on learning experience for her students which facilitated her teaching experience, as this quote states:

After learning AI using the e-book, students can carry out programming exercises based on the coding platform embedded in this e-book. This saves the time of programming environment configuration and also reduces the difficulty of going through the process. This is very important for beginner students and teachers.

Another teacher mentioned that the e-book was very easy to use since it provided hints about which sections are appropriate for each specific age, as well as some interactive quizzes that students can take to assess their knowledge, as follows:

The age appropriateness guidelines with the e-book facilitated our teaching process in terms of which topics we should focus on for each age category. This feature is very useful. I also like the quizzes, they are designed easily and they are very important to assess the students’ knowledge after finishing each learning concept.

PU was predicted significantly by PEOU (H1) (β = 0.835, *p* < 0.01) with 77.4% of the total variance explained. Meanwhile, attitude was predicted significantly by PEOU (H2) (β = 0.579, *p* < 0.01) and PU (H3) (β = 0.343, *p* < 0.01) with 83% of the total variance explained. The effect of PEOU on attitude was greater than PU, which implied that more work is needed to improve teachers’ perceived ease of use. This could be achieved by further enhancing the design of the open and interactive e-book. In terms of ease of use and usefulness, one teacher said that:

Several stories are included in this open and interactive AI e-book which helped students to understand the concept of AI easily, as they are very vivid and relevant to students’ daily life.

On the other hand, one teacher also mentioned that the open and interactive e-book was not friendly for mobile device users, as follows:

I usually read books on my mobile phone, but for this e-book, it was not friendly for me. For example, when I zoom in on the text, the page display gets messed up on the phone.

Hypotheses 4 and 5 refer to the relationship between continuance intention and perceived usefulness (H4) and attitude (H5). Intention to use the interactive e-book in this study was jointly predicted by perceived usefulness (H4) (β = 0.381, *p* < 0.01) and attitude (H5) (β = 0.536, *p* < 0.01). These variables explained 81.3% of the variance on intention to use (R^2^ = 0.813, Coefficient of determination). Among these relations, the attitude had a major influence on teachers’ behavior intention to use the open and interactive e-book. Moreover, attitude was more important than usefulness in determining behavioral intention to use the interactive AI e-book. When teachers were asked about their motivation for having the high intention of using this book in the future, one teacher mentioned the importance of openness and how it builds his confidence and saves his time, as follows:

This e-book is regarded as OER. This provides us opportunities to retain, revise, remix, reuse and redistribute the content, hence saving the time of preparing our teaching materials and being more confident to keep updating the content according to our needs, based on others’ views and inputs.

Intention to use the interactive e-book in this study was jointly predicted by perceived usefulness (H4) (β = 0.381, *p* < 0.01) and attitude (H5) (β = 0.536, *p* < 0.01). For instance, some of the primary school teachers were reluctant to use the e-book due to that some interactive functionalities were difficult for their students, as follows:

Since I am a primary school teacher, the embedded coding platform based on Python is too difficult for my primary school students. Visual programming platforms, such as Scratch, are more proper for them. Unfortunately, visual programming platforms are not embedded in this e-book which affects its usefulness.

**Fig. 3:**
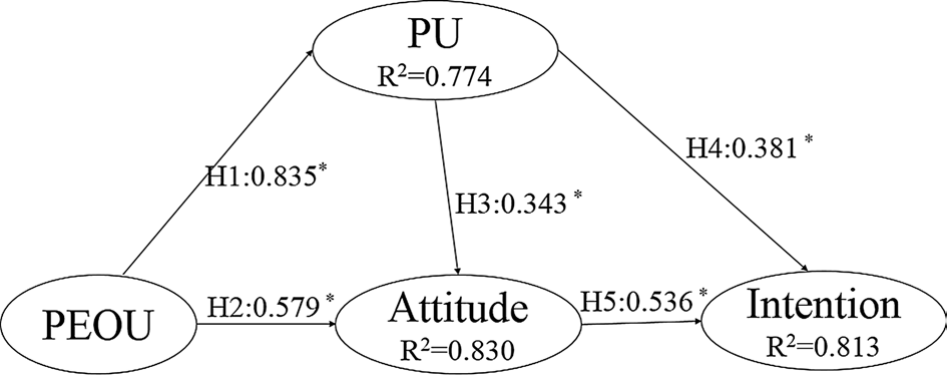
Result of the structural model; * denotes significant difference *p* < 0.01

Figure 3 summarizes the obtained findings related to the five hypotheses.

## Discussion

The purpose of this study was to identify the factors affecting teachers’ perception of the open and interactive e-book adoption based on TAM. The findings of the quantitative phase revealed that perceived ease of use and usefulness predicted k-12 teachers’ intention to use the open and interactive e-book, where attitude was a mediating variable. The qualitative data discussing teachers’ perceptions towards the e-book provided supplemental insights into the quantitative findings. The results also indicated that perceived ease of use is a strong indicator of perceived usefulness, which means that the more likely teachers perceive the open and interactive e-book to be easy to use, the more likely they will perceive it to be useful. This suggests that researchers should put more attention on the design process of e-books. Similarly, Jin (2014) found that the ease of use of e-books is significantly correlated with usefulness. Specifically, the obtained interview results showed that teachers found the e-book easy to use, especially the guidelines about age appropriateness, which helped them to efficiently use it in their teaching methods. The teachers, on the other hand, reported that the designed e-book is not friendly to use on their mobile devices. Therefore, it is suggested that researchers can provide instructional guidance which can help teachers to use e-books in classrooms, since some of them might not be familiar with e-books or have low ICT competencies that limit their use of e-books. Additionally, when designing e-books, designers and developers should keep in mind their usability on mobile devices.

The findings also revealed that perceived ease of use has a significant impact on attitude. That is, if an e-book is easy to use, teachers will have a positive attitude towards using it. Specifically, qualitative findings showed that the teachers reported that the developed e-book can be easily reused due to its openness feature, hence they were very motivated to use it for teaching AI. In line with this, Tang et al. (2020) also reported that the perceived ease of use of OER impacted the teachers’ perceived usefulness of using them, as well as their attitudes towards them. Therefore, it is suggested that researchers focus on designing open e-books which can help in re-using them and even updating them by others. Additionally, based on the interview results, the teachers also revealed that some interactive functionalities of the e-books were not easy to use, such as the embedded coding platform. They, therefore, recommended using visual programming software, such as Scratch (Maloney et al., 2010) and App Inventor (Patton et al., 2019) which are more appropriate for primary and secondary students. In this context, Aldosari et al. (2018) recommended that to increase the acceptance of information systems in education, researchers should consider students’ individual differences, such as gender and age. Therefore, when designing interactive e-books, the interactivity functionalities should be personalized according to teachers’ or students’ differences (e.g., age).

The obtained results further revealed that perceived usefulness, perceived ease of use, and attitude as mediating variables were positively associated with the continuance intention of using the developed e-book. Specifically, the teachers mentioned that the storytelling format of the content was very useful in engaging students in learning AI which made them motivated to continue using the e-book. Mohd (2008) stated that storytelling is a very useful feature to engage students in learning a given course. The teachers also stated that the different interactive functionalities within the e-book like the possibilities of students to assess their knowledge related to AI using quizzes, as well as interacting with their peers made the learning process more engaging and facilitated their teaching task.

## Conclusion, limitation and future work

This study aimed to develop an open and interactive e-book based on four interaction levels. It then examined the teachers’ continuance intention to use it based on TAM to promote the sustainable development of e-books. The results showed that teachers have a positive perception of the usefulness of this interactive e-book. Additionally, the teachers’ perceived ease of use had a significant influence on their perceived usefulness and on their attitude toward using the open and interactive e-book. Moreover, their perceived usefulness positively affected their attitude and intention towards using the open and interactive e-book. Meanwhile, teachers’ attitudes also had a positive effect on the intention to continuance using the open and interactive e-book.

This study does not only contribute to the existing studies to some extent but also helps researchers and participants obtain a deeper understanding of user behaviors in the acceptance of open and interactive e-books. It explores the relationships between different technology acceptance variables and explains how openness and interactive features might impact those variables when adopting open and interactive e-books by teachers. Additionally, this study should inform the development of e-books according to teachers’ feedback.

It should be noted that this pilot study has several limitations. First, this study had a limited sample size (only 57 participants). Additionally, external variables, namely openness, and interaction, which are the main features of this e-book were not considered as external variables in TAM. Thus, it is necessary to collect more data for analyzing the correlation between the two external variables (openness, interaction) and the four constructs of TAM. Additionally, this study only used users’ questionnaire data without considering their behavioral data within the open and interactive e-book. Finally, all participants were teachers, and no students were involved in this study to investigate their acceptance of using this e-book which may reflect a self-selection bias (Roca et al., 2006). However, despite these limitations, this study provided a solid ground for investigating teachers’ technology acceptance of open and interactive e-books for K-12 students.

In addition to considering the above limitations, future work could focus on making this open and interactive e-book smarter, by collecting students’ interaction data and analyzing them in real-time through a learning analytics system to provide personalized content, as well as instant feedback and recommendations when needed (i.e., the use of AI to enhance learning AI).

## Data Availability

The datasets generated and/or analyzed during the current study are not publicly available due to privacy reasons, but are available from the corresponding author on reasonable request.

## Abbreviations

OER: Open Educational Resources
OEP: Open educational practices
MOOCs: Massive open online courses
PU: Perceived usefulness
PEOU: Perceived ease of use
Att: Attitude
Int: Continuance Intentions
